# New Appliances and Things Medical

**Published:** 1896-09-26

**Authors:** 


### NEW APPLIANCES AND THINGS MEDICAL.

[We shall be glad to receive, at our Office, 428, Strand, London, W.C., from the manufacturers, specimens of all new preparations and appliances, which may be brought out from time to time.]

### "MAGGI'S CONCENTRATED CONSOMMÉ," FRENCH SOUPS AND SOUP ESSENCE.

(Cosenza and Co., 95-97, Wigmore Street, W.)

Messrs. Cosenza, who are the agents for the above preparations, have forwarded us samples for our inspection and trial. The concentrated consommé is really what it claims to be, namely, a perfectly clear soup, and not a beef tea or broth. In boiling water the concentrated preparation completely dissolves, showing no coagulum on further boiling without the addition of acetic acid. In its concentrated form it contains a very small percentage of water, consisting almost entirely of peptones and extractive of solid meat—in fact, so concentrated is it that the contents of a single capsule, about the size of a revolver bullet, is sufficient to make three-quarters of a pint of excellent consommé. The method of packing is highly ingenious, the concentrated consommé is poured into a mould or tube of gelatine, while the latter is again packed in a little capsule of the same substance. The two halves of the capsule, which fit one within the other, can be pulled apart, and the inner tube removed. The latter with its contents is thrown into a cup of boiling water and the consommé is ready for use. It requires no additional flavouring, and is a most appetizing preparation. In addition to the valuable qualities of concentration and pleasantness of flavour, Maggi's consommé is well tolerated by those of delicate digestion, and appears to be possessed of good nutritive qualities. But perhaps its most remarkable characteristics is its extraordinary cheapness. Boxes containing ten capsules, or sufficient for making more than seven pints of soup, can be purchased for 1s. 8d. Of the other varieties of concentrated soups prepared by the same firm we have only space to mention that they are about thirty in number; thus affording an almost infinite variety of really excellent soups at a price which is within the reach of almost all. Both for invalids and those in health this new departure in the way of ready-made soups will form an inestimable boon, and we do not hesitate to recommend our readers to give them a trial.

